# Evaluation of antioxidant activity and heavy metals content in licorice (*Glycyrrhiza glabra* L.) growing wild in Armenia

**DOI:** 10.1016/j.heliyon.2023.e22442

**Published:** 2023-11-17

**Authors:** H.R. Petrosyan, A.A. Nigaryan, H.A. Hovhannisyan, A.M. Soloyan, V.V. Vardapetyan, A.I. Martiryan

**Affiliations:** Yerevan State University, 1 A. Manoogian Street, 0025, Yerevan, Armenia

**Keywords:** Licorice, Antioxidants, Flavonoids, Anthocyanins, Tannins, Heavy metals

## Abstract

In this study, for the first time an analysis of the metal content in extracts obtained from licorice roots grown in the forests of five different regions in Armenia was conducted. Our findings indicated that the concentrations of metals in the extracts did not exceed the permissible limits set by regulatory standards. Furthermore, we investigated the quantitative composition of flavonoids, tannins, and anthocyanins in the licorice roots, which had not been previously studied. Our results revealed that the composition of these substances is significantly influenced by the soil characteristics of the region. To assess the antioxidant properties of the licorice extract, we employed the approach known as the kinetics of competitive reaction method. Our study successfully demonstrated that the extract derived from the roots of the licorice plant, collected from all five regions under study, exhibited notable antioxidant properties.

## Highlights

• Assessment of the concentrations of some heavy metals in licorice roots harvested from five regions of Armenia were done.
• Heavy metal concentrations in licorice root were within acceptable limits.
• The Kotayk and Tavush regions had the lowest combined heavy metal content.
• Investigation of antioxidant properties of licorice roots growing in five regions of Armenia were done.
• The Gegharkunik region showed the highest antioxidant activity.

## Graphical abstract

**Figure:**
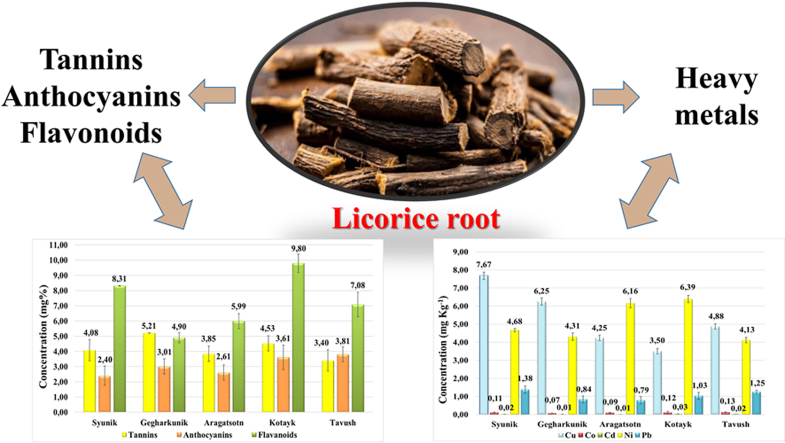

## 1 Introduction

Currently, there is a great interest in pharmaceuticals, nutritional supplements, cosmetics and other products made from natural raw materials. The use of such raw materials may lead to a limitation of the use of synthetic materials in these areas. Plants are a natural, valuable source of a certain group of chemical substances that are traditionally used in the production stages of new drugs, cosmetics, food additives, detergents and other products [[1], [2], [3]]. These substances are called biologically active due to their pharmacological or toxicological effects on human or animals [[4], [5], [6]]. They are synthesized in plants as secondary metabolites and play an important role in protecting plants from biotic or abiotic stress [7].

Clinical medicine research is now focusing on using natural substances and medicinal plants to treat ailments. There is a lot of interest in the quest for herbal remedies made from plants in order to find novel antioxidant and antibacterial compounds [8,9]. One of the most popular ancient herbs used in traditional medicine is licorice (*Glycyrrhiza glabra* L.) [10]. Glycyrrhizin is a key component of licorice's root. Since ancient times, glycyrrhizin has been utilized in a number of medicinal uses in addition to being a sweetener [11,12]. The ability of licorice to create a wide range of secondary metabolites is what gives it its medicinal value.

According to recent studies, flavonoids, anthocyanins, tannins, triterpenes and polysaccharide are among the most significant bioactive constituents of licorice [13,14]. They have been shown to have anti-inflammatory, anti-tumor, anti-microbial, antiviral, anti-inflammatory, antidiabetic, immunoregulatory, hepatoprotective, neuro-protective, and antioxidant effects. They also have functions similar to those of adrenal cortical hormones [[15], [16], [17], [18]].

The investigation of antioxidant and antiradical compounds is currently a subject of extensive research. The chemical composition of these substances directly influences the mechanism of oxidation processes [[19], [20], [21]]. Given the widespread use of antioxidants in pharmaceuticals, cosmetics, and food industries, understanding their function is both theoretically important and practically useful [[22], [23], [24]]. The exploration of plants, fruits, and vegetables as a source of antioxidants has become a priority in academic institutions [25]. In addition to the above-mentioned points, plants have the ability to mitigate the detrimental effects of heavy metals when they are present in soils in natural amounts [26]. Nevertheless, elevated levels of heavy metals can negatively impact the cellular and physiological functions of plants. The extent of harm depends on the concentration of these components in both soil and plant life [27]. Furthermore, it is recognized that the quantitative content of various metals in plants is influenced by their geographical location [28].

From this point of view, based on the importance of the above mentioned, quantitative content of the main antioxidants and some heavy metals (Co, Cd, Cu, Ni, Pb) of licorices root extracts growing in Armenia (five regions: Syunik, Gegharkunik, Aragatsotn, Kotayk, Tavush) was studied in this work. To obtain extracts, dry roots of licorice have been used. GOST 24027.2–80, 13399-89, 32709-2014 methods [29,30] were used to determine the quantitative content of flavonoids, anthocyanins and tannins. Heavy metals were determined by using the method of atomic absorption spectroscopy. Antioxidant properties of extracts were examined by studying the kinetics of the competitive reaction method [31,32]. This method assesses antioxidant activity by examining the kinetics of the competitive reaction between the hydroxyl radicals of the test sample and *p*-nitrosodimethylaniline (PNDMA).

The rationale behind this research stems from the novel application of a previously unexplored approach to investigate the antioxidant properties of the licorice root extract. Notably, the specific use of this approach to assess the antioxidant properties of the plant species within the geographical boundaries of Armenia adds further significance to the study. Moreover, the lack of existing research examining both the antioxidant properties and metal composition of this particular plant species within the region underscores the importance of conducting this investigation.

## 2 Materials and methods

Roots of licorice were collected at the end of May 2022 from the forests (far from motor roads and settlements; 20 samples for each region) of Syunik, Gegharkunik, Aragatsotn, Kotayk, Tavush regions of Armenia.

### 2.1 Chemicals and regents

The following materials were used for the experiments: potassium permanganate (Merck, Darmstadt, Germany, purity≥99 %), indigo carmine (Merck, Darmstadt, Germany, purity≥85 %), hydrochloric acid (37 %, Merck, Darmstadt, Germany), aluminum chloride (Sigma Aldrich, USA, purity≥99 %), rutin (Sigma Aldrich, USA, purity≥95 %), acetic acid (Sigma Aldrich, USA, purity≥99 %), ethanol (Sigma Aldrich, USA, purity≥95 %), nitric acid (65–67 %, Sigma Aldrich, USA), 1000 mg l^−1^ monoelemental standard (Sigma Aldrich, USA) and high purity argon (99.998 %, Airgas, Denver, USA).

### 2.2 Sample preparation for determination of tannins

The quantitative determination of tannins in roots of licorice was carried out according to GOST 24027.2–80. Initially, licorice roots (2 g) were weighed into a flat-bottomed flask with a capacity of 500 mL. To this, distilled water (250 mL) was added, and the mixture was boiled for 30 min with a reflux condenser under stirring conditions. After cooling, the extract was filtered using cotton wool. Subsequently, the extract (25 mL) was transferred to a 750 mL flask, and distilled water (500 mL) as well as indigo carmine solution (25 mL) were added. The resulting solution was titrated with KMnO_4_ (0.1 N) until it exhibits golden hue.

The process was repeated five times for each sample to account possible deviations.

### 2.3 Sample preparation for determination of flavonoids

The quantitative determination of flavonoids in licorice roots of was carried out according to GOST 13399-89. Roots (1 g) were crushed and placed in 200 mL flask. To this, 70 % ethyl alcohol was added (100 mL). The contents of the flask were weighed, and the mixture was connected to a reflux refrigerator and boiled for 30 min. After cooling, the mixture was re-weighed (solvent has been added to restore the weight) and filtered through a paper filter. The first 20 mL of mixture was discarded. Subsequently, the extract (1 mL) was transferred into a volumetric flask with a capacity of 25 mL. To this, pre-prepared alcoholic solution of 2 % AlCl_3_ (5 mL) was added, and the volume was adjusted to the mark with alcohol. After 30 min, the optical density was measured at a wavelength of 410 nm. As a reference solution, extract (2 mL) + concentrated acetic acid (0.2 mL in 24.8 mL of alcohol) were used. In parallel, under the same conditions, the optical density of a standard sample of rutin was measured, using 95 % ethyl alcohol as a reference.

The process was repeated five times for each sample to account possible deviations.

### 2.4 Sample preparation for determination of anthocyanins

The quantitative determination of anthocyanins in licorice roots was carried out according to GOST 32709-2014. Initially, plant material (0.3 g) was weighted and sieved with a 1 mm grid, then transferred to a 250 mL flat-bottomed flask. To this, 1 % HCl (100 mL) was added, and the mixture was refluxed on a water bath for 15 min. After cooling, the extract was filtered through cotton wool. An additional 1 % HCl (100 mL) was added, and the extraction process was repeated for another 15 min. The resulting extract was again filtered through cotton into a 250 mL flask and the volume of the flask was adjusted to the mark by washing the cotton with 1 % HCl. The optical density of the solution was measured at a wavelength of 510 nm.

The process was repeated five times for each sample to account possible deviations.

### 2.5 Measurement of antioxidant properties

Spectroscopic measurements were conducted using T60 UV-VIS spectrometer (PG Instruments Ltd, United Kingdom).

The antioxidant activity of licorice root extract was assessed (the process was repeated three times) using the PNDMA test. Licorice roots were subjected to extraction using the maceration method. Specifically, 5 g of root material was immersed in distilled water, maintaining a stable temperature of 70–75 °C for 40 min. The resulting solutions were then filtered, and this process was repeated multiple times to ensure thorough extraction.

In this approach, the kinetics of the competitive interaction between hydroxyl radicals and PNDMA are studied to evaluate the antioxidant characteristics. Hydroxyl radicals, generated when hydrogen peroxide is exposed to UV light at 313 nm, react with PNDMA, leading to the decolorization of the dye. Using a photometric approach, the rate of the reaction between hydroxyl radicals and PNDMA is estimated from the absorbance at 440 nm. This process is influenced by the addition of extract because hydroxyl radicals and extract chemicals compete with each other, resulting in a slower decolorizing of PNDMA. The following equation can be used to determine the rate constant of the reaction between extract compounds and hydroxyl radicals by examining the rate of dye decolorization:

(1) $$K_{\text{OH}+\text{extract}}=1.25\cdot 10^{10}\cdot \frac{\left[\text{PNDMA}\right]}{\left[\text{Extract}\right]}\cdot \left(\frac{W_{1}}{W_{2}}-1\right)$$

where [Extract] and [PNDMA] are molar concentrations of extract and PNDMA, respectively. 1.25∙10^−10^ is the rate constant of reaction between hydroxyl radical and PNDMA (l mol^−1^∙s^−1^). W_1_ and W_2_ are the slopes of plots of PNDMA absorption versus time of hydrogen peroxide radiation in the absence and presence of surfactants, respectively.

### 2.6 Sample preparation for determination of Co, Cd, Cu, Ni, Pb heavy metals

PG-990 Atomic Absorption Spectrometer (Leicestershire, United Kingdom) equipped with deuterium background correction, eight hollow cathode lamps, deuterium lamp, horizontally heated platform graphite tube was used for Co, Cd, Cu, Ni, Pb metals determination.

The samples for heavy metal determination were prepared as follows: in a refractory crucible roots (12 g) were weighted and heated until black ash was formed. This was placed in a muffle furnace with the following sequence: 250 ^o^С - 1 h, 300 ^o^С - 1 h, 350 ^o^С - 1 h, 400 ^o^С - 1 h, 450 ^o^С - 3 h. After cooling, a milky white mass with gray granules was observed which was treated with concentrated nitric acid (5 mL) and placed in a muffle furnace (after nitric acid evaporation process) at a temperature of 450 ^օ^C until the entire mass acquired a milky white color. After, the samples were dissolved in 1 % nitric acid (10 mL), filtered, washed and diluted in a 25 mL volumetric flask. Based on the technical conditions of the AAS PG 990, the obtained solutions were additionally diluted 25 times with 0.1 % nitric acid.

The experiment for each sample was repeated three times and mean values were calculated by AAWin Pro software.

Analytical and heating programs for all determinations are shown in Table 1, Table 2.

**Table 1:** Analytical programs for Co, Cd^,^ Cu, Ni, Pb metals determination.

Metals	Analytical line (nm)	Sample size (μl)	Acidity	Background
Co	240.7	10	0.1 % HNO_3_	D2
Cd	228.8	10	0.1 % HNO_3_	D2
Cu	324.7	10	0.1 % HNO_3_	None
Ni	232.0	10	0.1 % HNO_3_	D2
Pb	283.3	10	0.1 % HNO_3_	None

**Table 2:** Heating program for Co, Cd^,^ Cu, Ni, Pb metals determination.

Metals	Stage	Temperature, ^o^C	Ramp, s	Hold, s
Co	1	70	5	10
	2	100	10	10
	3	800	10	10
	4	2000	0	3
	5	2100	1	2
Cd	1	90	5	10
	2	120	5	10
	3	500	5	10
	4	1800	0	3
	5	1900	1	2
Cu	1	70	5	10
	2	110	10	10
	3	600	10	15
	4	2100	0	3
	5	2200	1	2
Ni	1	80	5	10
	2	110	10	15
	3	800	15	10
	4	2000	0	3
	5	2100	1	2
Pb	1	70	10	10
	2	110	10	10
	3	450	10	15
	4	1800	0	3
	5	1900	1	2

## 3 Results and discussion

### 3.1 Quantitative determination of tannins, anthocyanins, flavonoids

**Fig. 1:**
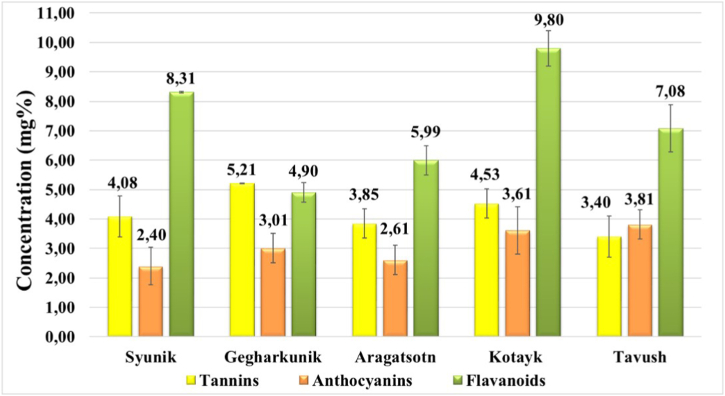
Quantitative content of tannins, anthocyanins, flavonoids in terms of dry raw materials in licorice's roots collected from five regions (Syunik, Gegharkunik, Aragatsotn, Kotayk, Tavush) of Armenia.

The quantitative assessment of biologically active compounds in licorice root samples collected from five regions of Armenia revealed the presence of anthocyanins, tannins, and flavonoids. Fig. 1 illustrates that flavonoids were the predominant compounds across all samples. The flavonoid content in licorice roots collected from the forests of Syunik, Gegharkunik, Aragatsotn, Kotayk, and Tavush regions was measured to be 8.31 ± 0.03 mg%, 4.90 ± 0.03 mg%, 5.99 ± 0.5 mg%, 9.80 ± 0.6 mg%, and 7.08 ± 0.8 mg%, respectively. Notably, licorice roots obtained from the forests of Syunik, Kotayk, and Tavush exhibited relatively higher levels of flavonoids, potentially attributed to the presence of more fertile soils. The quantitative content of anthocyanins and tannins remained consistent across all samples, with an average tannin quantity of approximately 4 mg% and anthocyanin quantity of approximately 3 mg%.

It is worthy to note that mg% is defined as the number of milligrams of the substance of interest contained in 100 g of the investigated sample.

Comparing the regions, it can be concluded that the soil in Kotayk is more conducive to the accumulation of substances with antioxidant properties in the roots of the plant. This observation can be attributed to the specific composition of the soil prevalent in that particular region.

Upon a comparative analysis with existing data available in scientific literature, it is evident that the concentration of flavonoids within the roots of the examined plant is notably lower when contrasted with the levels found in the roots of the same plant species obtained from different geographical regions. For instance, the investigations conducted in the research works [33,34] insights into the quantification of flavonoids in plants native to Khorasan and Fars provinces in Iran. While Iran shares a border with Armenia, the empirical data obtained indicates that the plant species flourishing within Armenia's territory exhibits a more pronounced capacity for flavonoid accumulation.

A separate study endeavors to examine the quantitative presence of flavonoids within the composition of select plant species endemic to the Armenian territory, including *Laurus nobilis*, *Ocimum basilicum*, and *Hypericum perforatum*. Notably, this investigation also reveals that the concentration of flavonoids within the roots of the focal plant under study is lower in comparison to the findings reported in the work [35].

Furthermore, it was found out that the quantification of tannins within licorice roots closely approximates the tannin levels recorded in the *Polygonum hydropiper* collected from the Gegharkunik region of Armenia [36].

### 3.2 Quantitative determination of Co, Cd, Cu, Ni, Pb heavy metals

The analysis of copper, cobalt, cadmium, nickel and lead heavy metals in licorice roots was conducted using an atomic absorption spectrophotometer, with all necessary safety precautions implemented to prevent potential contamination.

The concentrations of copper were determined to be 7.67 ± 0.21 mg kg^−1^, 6.25 ± 0.20 mg kg^−1^, 4.25 ± 0.15 mg kg^−1^, 3.50 ± 0.15 mg kg^−1^, and 4.88 ± 0.15 mg kg^−1^ in the samples obtained from the forests of Syunik, Gegharkunik, Aragatsotn, Kotayk, and Tavush regions, respectively. All these values fall below the permissible limit (10 mg kg^−1^) for copper [37].

It is worth noting that the highest amount of copper was observed in the Syunik region, which aligns with expectations due to the presence of a copper-molybdenum factory operating in that area. Consequently, the factory's activities naturally exert an influence on the copper content within the soil.

The measured concentrations of cobalt were found to be 0.11 ± 0.02, 0.07 ± 0.01, 0.09 ± 0.03, 0.12 ± 0.05, 0.13 ± 0.01 mg kg^−1^․ According to various sources [38,39], the maximum allowed concentration for cobalt is in the range of 0.1–0.2 mg kg^−1^․ Thereby, in the results obtained, excesses as such were not observed.

The cadmium concentrations were determined as 0.02 ± 0.007, 0.01 ± 0.003, 0.01 ± 0.003, 0.03 ± 0.003, 0.02 ± 0.003 mg kg^−1^, respectively. The permissible quantity of Cd in plants is 0.02 mg kg^−1^ [37]. This means that the roots collected from mentioned regions are ecologically clean from Cd ions.

**Fig. 2:**
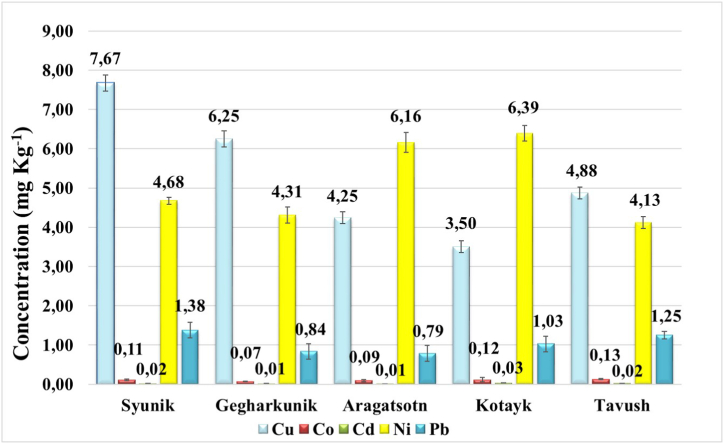
Determination of Cu, Co, Cd, Ni and Pb heavy metals in licorice's roots collected from five regions (Syunik, Gegharkunik, Aragatsotn, Kotayk, Tavush) of Armenia.

The concentrations of nickel and lead were found to be 4.68 ± 0.09, 4.31 ± 0.2, 6.16 ± 0.25, 6.39 ± 0.20, 4.13 ± 0.15 mg kg^−1^ and 1.38 ± 0.20, 0.84 ± 0.20, 0.79 ± 0.20, 1.03 ± 0.20, 1.25 ± 0.10 mg kg^−1^, respectively. Both of these metals were within proper limits of 10 mg kg^−1^ and 2 mg kg^−1^, respectively [37]. The summarized results are presented in Fig. 2.

It is noteworthy to highlight that the Syunik, Gegharkunik, and Tavush regions exhibit the highest concentration of copper among the analyzed heavy metals, while in the Aragatsotn and Kotayk regions, the amount of nickel surpasses that of copper.

Overall, no excessive levels of heavy metals were detected in the collected samples from any examined region of Armenia. However, when comparing the different regions, it can be observed that the combined content of heavy metals in Kotayk and Tavush is the lowest, indicating that the soil in these areas displays the least contamination by the aforementioned metals.

In the work [40], an analysis is conducted to determine the levels of specific heavy metals in the roots of licorice plants cultivated in Egypt. The findings indicate that, for instance, the copper content in these roots is quantified at 20 mg kg^−1^, a measure approximately fivefold greater than the mean copper concentration observed in licorice plants collected from five distinct regions across Armenia.

In the study [41], detailed findings pertaining to the metal content in *Chamerion angustifolium (L.) Holub* growing in Armenia are elucidated. Notably, in comparison with these data, it is observed that the copper levels are commensurate. Conversely, cobalt is nearly double in the studied plant, whereas cadmium exhibits higher concentrations in *Chamerion angustifolium (L.) Holub.* The most significant disparities are evident in the context of nickel and lead, where the levels of these metals in licorice roots are substantially greater by several times.

Long-term consumption of plants containing heavy metals within acceptable limits is typically regarded as safe for human health. Nevertheless, it's important to consider several factors that can influence the potential health effects [37]. For instance, the form of heavy metals in the food and how easily they can be absorbed by the body can impact their health implications. Maintaining a balanced and diverse diet can help reduce the potential risks associated with the long-term consumption of heavy metals. By consuming a wide range of foods, the influence of any one specific source can be diluted. Furthermore, certain individuals, such as pregnant women, infants, and those with specific health conditions, may be more susceptible to the adverse effects of heavy metals. Therefore, it's crucial to remain attentive to guidelines and regulations that monitor and limit heavy metal content in food. Continuous research and assessment help refine these standards to ensure they adequately protect public health.

When considering the sources of heavy metals in plants in Armenia, as in many other regions, it can result from a variety of natural and anthropogenic factors. Armenia, being a geographically rocky country, is susceptible to the gradual release of heavy metals into the soil due to the weathering of rocks over time. These metals are subsequently absorbed by plants. Additionally, Armenia's industrial and mining activities release heavy metals into the environment through emissions, wastewater discharge, or the improper disposal of industrial waste. It's worth noting that the regions under examination are heavily involved in agricultural practices, including the use of fertilizers, pesticides, and herbicides, all of which can contribute to the accumulation of heavy metals in the soil.

### 3.3 Antioxidant properties

**Fig. 3:**
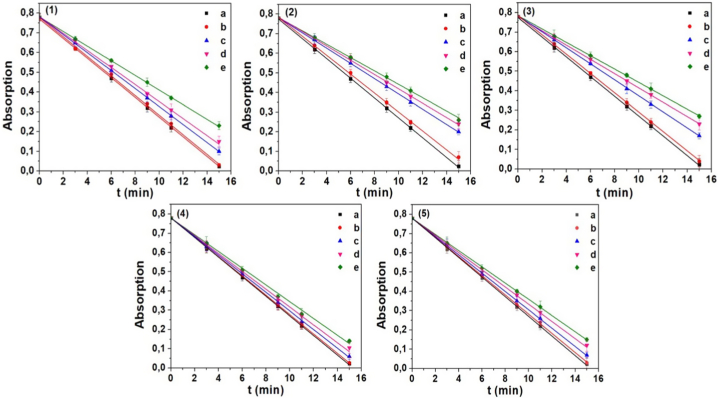
Absorption of PNDMA vs hydrogen peroxide radiation time at different concentrations of licorice root extract collected from (1) Syunik, (2) Gegharkunik, (3) Aragatsotn, (4) Kotayk, (5) Tavush regions. Molar concentration of the extract was a-0, b-0.02, c-0.04, d-0.10, e−0.16 g l^−1^.

Fig. 3 illustrates the relationship between the absorption of PNDMA and the radiation period of hydrogen peroxide in the presence and absence of the investigated ex0tract. It is evident that as the concentrations increase in all regions, the rate of the competitive reaction between hydroxyl radicals and PNDMA (decolorization) decreases significantly. This indicates that the extract reacts with hydroxyl radicals, resulting in a slower competitive reaction between PNDMA and hydroxyl radicals.

This observation suggests that the plant extract possesses notable antioxidant properties. Furthermore, this effect is observed consistently across all regions for the extract derived from plant roots. As a comparison, experiments were also conducted without the extract, considering an extract concentration of zero.

**Fig. 4:**
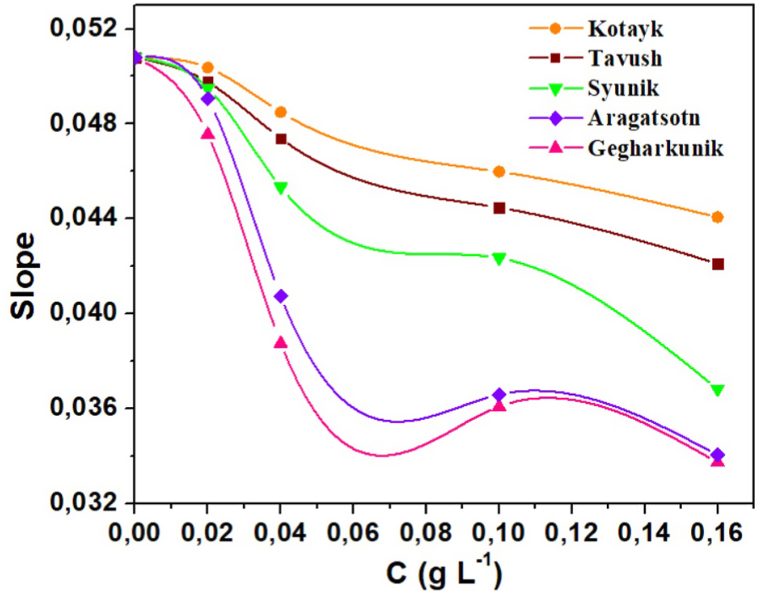
Slopes of plots presented in Fig. 3 vs concentration of the extract.

The rate of the hydroxyl radical-PNDMA reaction is determined from the slopes of the plots. Fig. 4 depicts the relationship between the slopes and the extract concentration. The rate constants of the extract-hydroxyl radical reaction are derived by plotting the reaction rate against the surfactant concentration using Equation (1). The obtained results are presented in Table 3.

**Table 3:** Rate constants of the reaction between the extract of licorice root from five different regions of Armenia and hydroxyl radicals.

Region	Rate constant, mol^−1^ l s^−1^
Syunik	1.20 10^7^
Gegharkunik	3.18 10^7^
Aragatsotn	2.75 10^7^
Kotayk	5.21 10^6^
Tavush	5.97 10^6^

The detection process involves extracting the corresponding slope from the decolorization reaction curves for each concentration, and subsequently establishing a relationship between the slope and the concentrations. Each dependence curve exhibits a breakpoint, which, in this case, can be considered as the concentration value of 0.067 g l^−1^ for all regions. At this concentration, the slope values are fixed and utilized in the corresponding Equation (1).

The results presented in Table 3 demonstrate the variations in the reaction rates between the investigated extract and hydroxyl radicals. Among the different regions, licorice root collected from the Gegharkunik region exhibited the highest rate of reaction. The reaction rates decreased in the following order: Gegharkunik > Aragatsotn > Syunik > Tavush > Kotayk. Notably, the reaction rates obtained from the Syunik, Gegharkunik, and Aragatsotn regions were relatively close to each other, and were tenfold or higher compared to the results obtained from the other two regions.

These findings highlight the significant influence of regional variations on the extract's reactivity with hydroxyl radicals. The observed higher reaction rates in Gegharkunik, Aragatsotn, and Syunik regions suggest the presence of certain constituents or environmental factors that enhance the extract's antioxidant properties. Further investigation into the specific compounds or environmental conditions responsible for these regional differences may provide valuable insights for optimizing the extract's applications in antioxidant-related research and potential therapeutic interventions.

The body's cellular molecules do not directly engage with overactive free radicals, but the hydroxyl groups found in these compounds facilitate interaction, effectively reducing the consumption of these free radicals without affecting the body's molecules. Furthermore, flavonoids, tannins, and anthocyanins found in plants enhance their antioxidant properties by counteracting free radicals, binding to metal ions, and obstructing enzymes responsible for free radical production. When we incorporate plant-based foods rich in these compounds into our diet, they extend similar antioxidant advantages to our bodies, aiding in the mitigation of oxidative stress and potentially reducing the risk of chronic illnesses.

Certainly, this study not only offers novel insights into the metal content, composition of bioactive compounds, and antioxidant properties of licorice extracts for the first time for different regions of Armenia but also underscores the significant implications of these findings in advancing our understanding of the region's agricultural potential and the broader field of natural product research.

## 4 Conclusions

Thus, for the first time some Cu, Co, Cd, Ni and Pb heavy metals content in licorice extracts from five regions (Syunik, Gegharkunik, Aragatsotn, Kotayk, Tavush) of Armenia was examined, with a focus on forest areas. It has been confirmed that the metal concentrations in the extracts were within acceptable limits.

Furthermore, the quantitative composition of flavonoids, tannins, and anthocyanins in licorice roots, an area not previously explored, was investigated. Licorice roots from Syunik, Kotayk, and Tavush forests exhibited relatively higher levels of flavonoids. All samples had consistent 4 mg% tannin and 3 mg% anthocyanin content.

The antioxidant properties of the licorice extract were assessed by utilizing the competitive reaction kinetics method. Remarkable antioxidant properties were successfully demonstrated in the extracts obtained from licorice roots across all five regions. The Gegharkunik region exhibited the highest level of antioxidant activity among the regions studied.

## Funding

This work was supported by the Science Committee of the Republic of Armenia [grant number 22YR-1D023].

## Data availability statement

Data will be made available on request.

## Additional information

No additional information is available for this paper.

## CRediT authorship contribution statement

**H.R. Petrosyan:** Writing – review & editing, Writing – original draft, Visualization, Supervision, Resources, Project administration, Investigation, Funding acquisition, Data curation, Conceptualization. **A.A. Nigaryan:** Validation, Formal analysis, Data curation. **H.A. Hovhannisyan:** Validation, Formal analysis, Data curation. **A.M. Soloyan:** Validation, Formal analysis, Data curation. **V.V. Vardapetyan:** Visualization, Project administration, Data curation, Conceptualization. **A.I. Martiryan:** Supervision, Methodology, Investigation, Conceptualization.

## Declaration of competing interest

The authors declare that they have no known competing financial interests or personal relationships that could have appeared to influence the work reported in this paper.
